# Simulation of Lake Victoria Circulation Patterns Using the Regional Ocean Modeling System (ROMS)

**DOI:** 10.1371/journal.pone.0151272

**Published:** 2016-03-31

**Authors:** Chrispine Nyamweya, Christopher Desjardins, Sven Sigurdsson, Tumi Tomasson, Anthony Taabu-Munyaho, Lewis Sitoki, Gunnar Stefansson

**Affiliations:** 1 Kenya Marine and Fisheries Research Institute, Kisumu, Kenya; 2 School of Engineering and Natural Sciences - Faculty of Physical Sciences, University of Iceland, Reykjavik, Iceland; 3 United Nations University Fisheries Training Programme, Marine Research Institute, Reykjavik, Iceland; 4 National Fisheries Research Institute, Jinja, Uganda; 5 The Technical University of Kenya, Nairobi, Kenya; University of California San Diego, UNITED STATES

## Abstract

Lake Victoria provides important ecosystem services including transport, water for domestic and industrial uses and fisheries to about 33 million inhabitants in three East African countries. The lake plays an important role in modulating regional climate. Its thermodynamics and hydrodynamics are also influenced by prevailing climatic and weather conditions on diel, seasonal and annual scales. However, information on water temperature and circulation in the lake is limited in space and time. We use a Regional Oceanographic Model System (ROMS) to simulate these processes from 1^st^ January 2000 to 31^st^ December 2014. The model is based on real bathymetry, river runoff and atmospheric forcing data using the bulk flux algorithm. Simulations show that the water column exhibits annual cycles of thermo-stratification (September–May) and mixing (June–August). Surface water currents take different patterns ranging from a lake-wide northward flow to gyres that vary in size and number. An under flow exists that leads to the formation of upwelling and downwelling regions. Current velocities are highest at the center of the lake and on the western inshore waters indicating enhanced water circulation in those areas. However, there is little exchange of water between the major gulfs (especially Nyanza) and the open lake, a factor that could be responsible for the different water quality reported in those regions. Findings of the present study enhance understanding of the physical processes (temperature and currents) that have an effect on diel, seasonal, and annual variations in stratification, vertical mixing, inshore—offshore exchanges and fluxes of nutrients that ultimately influence the biotic distribution and trophic structure. For instance information on areas/timing of upwelling and vertical mixing obtained from this study will help predict locations/seasons of high primary production and ultimately fisheries productivity in Lake Victoria.

## Introduction

Lake Victoria is thought to have formed about 400,000 years ago by down-warping of land between the two arms of the Great Rift Valley. Westward-flowing rivers were dammed by an upthrown crustal block, reversing their flow into the down warped land. During its geological history the lake topography went through changes ranging from its present shallow depression, to what may have been a series of much smaller swampy lakes that gradually became interconnected, lost their outlet to the west and were drained northwards by the Nile [1]. Geological cores taken from bottom sediments show that Lake Victoria has dried up completely at least three times since it formed [2]. Measured by surface area (68,000 km^2^), it is the largest fresh water lake in the tropics and second in the world. However, due to its shallowness, it is the 17^th^ largest among the world lakes by volume [2]. The lake is shared among Kenya (6%), Uganda (43%) and Tanzania (51%). The catchment is about 193,000 km^2^ extending to Rwanda and Burundi. About 33 million inhabitants in the basin within the three East African countries depend on the lake for transport, water for domestic and industrial uses and fisheries [3].

The lake plays an important role in modulating regional climate. Its thermodynamics and hydrodynamics are also influenced by climatic factors such as the Intertropical Convergence Zone (ITCZ), El Nino/Southern Oscillation (ENSO), complex orographic forcing, and the Indian Ocean zonal temperature gradient anomalies [4, 5] on diel, seasonal and annual scales [6]. The ITCZ that separates the northeast and southeast monsoons, crosses East Africa twice every year, once during March-April-May and again during October-November-December. This incursion and retreat of the ITCZ is responsible for the two main rainfall and dry seasons of the region. The rainy season from March through to May is commonly known as the ‘long rains’; the second rainy season of October through to December is called the ‘short rains’ [7]. The water budget is controlled mainly through precipitation over the lake surface, catchment inflow, controlled outflow at a hydroelectric dam on River Nile and evaporation [2, 8, 9]. The lake does have a season of deep vertical mixing when the lake becomes isothermal. During June and July the established thermocline breaks down under the seasonal onset of the south-east trade winds and for a brief period at the end of July the main body of the lake becomes isothermal with respect to depth. The thermocline most often occurs at 30–40 m depth. Complete mixing occurs once a year [10].

Lake Victoria is not physico-chemically homogenous. Much of the shoreline in the north and south is highly irregular. The northern shallow waters are intercepted by numerous islands. East and west of the lake, the basin rises over a thousand meters to highlands bordering the respective rift valleys, but to the north and south the watershed is less than 25 m above lake level [2]. Water quality varies spatially in Lake Victoria. Gulfs, near-shore areas adjacent to big human settlements and river mouth areas are relatively turbid and eutrophic [11]. The diverse topography/terrain, prevailing weather/climatic conditions as well as river inflows and outflows influence water circulation patterns. This in turn determines the temporal-spatial water quality which can be linked to the distribution of biota in the lake [11].

Previous hydrographic studies have explored water movements on a small scale either in space or time. Anyah and Semazzi (2009) [4] developed an idealized simulation of hydrodynamic characteristics of Lake Victoria. The authors relied on idealized (and real) bathymetry and uniform surface wind stress over a period of sixty days. MacIntyre (2014) [6] studied thermo-stratification and factors affecting horizontal exchange while Okely (2010) [11] evaluated processes affecting horizontal mixing and dispersion in the Nyanza Gulf of Lake Victoria.

To better understand annual and seasonal water circulation (currents) in Lake Victoria, a Regional Oceanographic Model System (ROMS) is developed. The model is based on real bathymetry, wind stress, surface heat fluxes, solar radiation and river inflow/outflow forcing. The ROMS includes accurate and efficient physical and numerical algorithms and several coupled models. It also includes several vertical mixing schemes [12], multiple levels of nesting and composed grids.

## Materials and Methods

**Fig 1:**
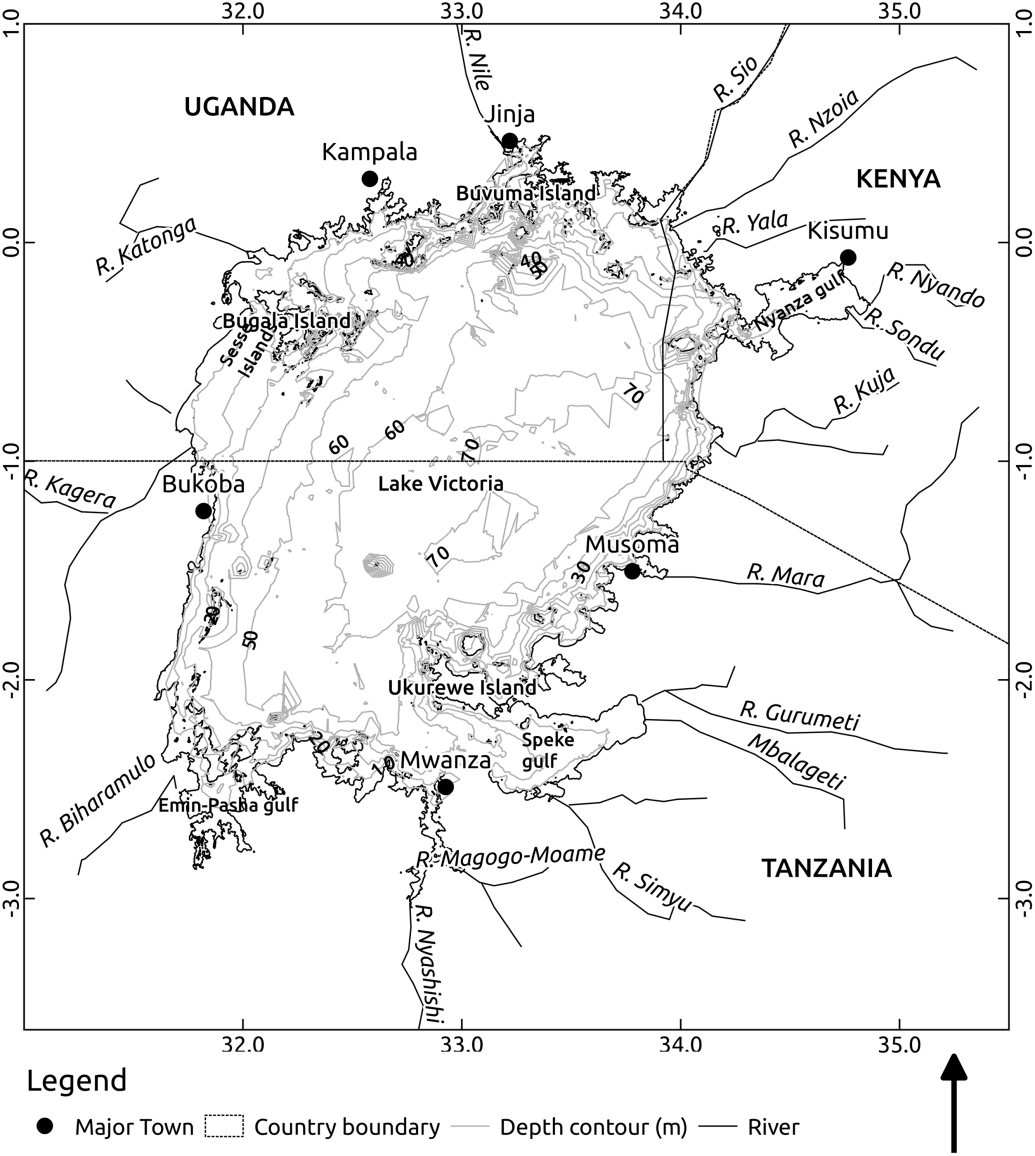
Map of Lake Victoria, its major rivers and bathymetry.

The Lake Victoria ROMS model extends from 31.5°—34.88° E and 3.05° S to 0.55° N (Fig 1). It has a horizontal grid resolution of 1.9 km. The total number of grid points is 209 and 216 in the longitudinal and latitudinal directions respectively. The fine grid resolution is chosen to adequately resolve processes within narrow gulfs, bays and indented coastlines. The model has 20 terrain-following vertical levels with a minimum depth set to 5 m. Preliminary model runs with fewer vertical levels did not realistically mimic observed temperature profiles failing to accurately predict the thermocline depth as result of averaging values over wide depth ranges. The model has surface stretching factor *θ*_*s*_ = 3 to maintain high resolution throughout surface layers of the model domain. *θ*_*b*_ is set to 1 to allow the influence of bathymetry on overlying layers. Here, *θ* is a refinement parameter that determines the magnitude of stretching of the vertical grid in either the surface (*θ*_*s*_) or bottom layers (*θ*_*b*_). The thermal expansion coefficient and background density values are estimated for fresh water at 24°C. A typical linear bottom drag coefficient [13, 14] is applied for the entire computation domain given the small depth range of the lake. Other model parameters are described in Table 1

**Table 1:** Basic model configuration.

Parmeter	Value	Description
Lm	209	number of points in longitude direction
Mm	216	number of points in latitude direction
N	20	number of vertical (sigma) levels
*h*_*max*_	80 m	maximum depth of the domain
*h*_*min*_	5 m	minimum depth of the domain
*θ*_*s*_	3	sigma coordinate surface stretching factor
*θ*_*b*_	1	sigma coordinate bottom stretching factor
Δ_*t*_	60 s	baroclinic time-step
Δ_*tf*_	20 s	barotropic time step
*r*	3.0 10^−4^ m^2^s^−1^	linear bottom drag coefficient
*Tcoef*	2.4725 10^−4^	Thermal expansion coefficient
*ρ*	997 kgm^−3^	Background density value

**Fig 2:**
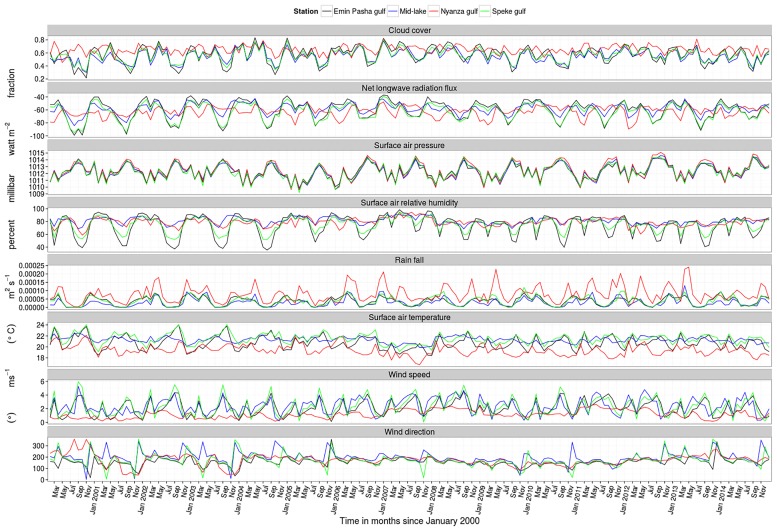
Evolution of different model forcing variables: cloud cover, net longwave radiation, surface air pressure, surface air relative humidity, rainfall, surface air temperature, wind speed and direction at selected locations in Lake Victoria from the year 2000–2014.

The bathymetry/topography (Fig 1) is derived from lake wide hydro-acoustic survey data. Meteorological surface forcing data are obtained from the European Centre for Medium-Range Weather Forecasts (ECMWF) ERA Interim, daily data set extracted at 3 hour time steps with 0.125° horizontal resolution from 1^st^ January 2000 to 31^st^ December 2014. Cloud cover, net longwave radiation flux, surface air pressure, surface air relative humidity, rain fall, surface air temperature, Surface v- and u-wind components 10 meters above the water surface and river runoff forcing fields (Fig 2) are implemented using the bulk flux algorithm [15]. The model is initialized with a uniform temperature of 24°C and no momentum on 1^st^ January 2000. Salinity is set to zero. Simulated spatial-temporal temperature trends are fitted to observation data collected during the 2000–2001 and 2005–2014 lake wide hydro-acoustic surveys. Modeled ($\hat{y}$) and observed (*y*) vertical temperature profiles are compared using the Root Mean Squared Error (RMSE).

$$\begin{array}{c} RMSE=\sqrt{\frac{1}{n}\sum_{i=1}^{n}{\left(y_{i}-{\hat{y}}_{i}\right)}^{2}} \end{array}$$ (1)

## Results

**Fig 3:**
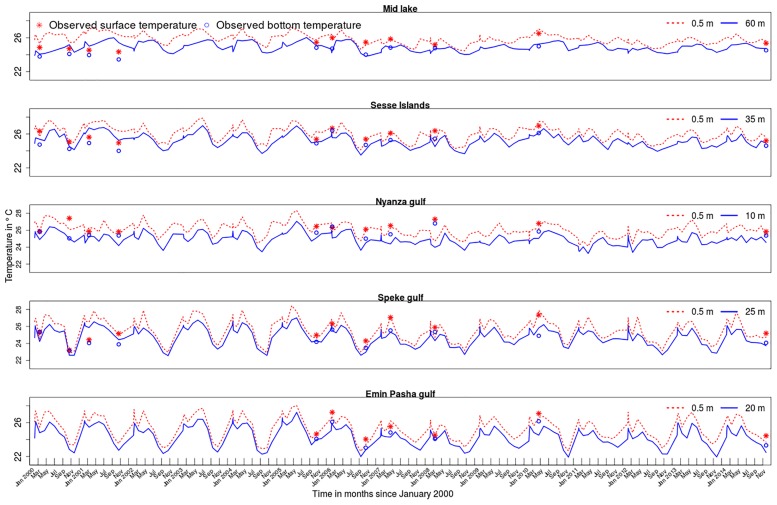
Modeled and observed surface and bottom temperature trends at selected sites in Lake Victoria.

Results from the Lake Victoria ROMS model show spatial and temporal variations in water temperature. Modeled temperatures largely agree with observations (Fig 3) except at the beginning of the model run in the deeper stations. Table 2 shows simulated temperature statistics at 5 selected sites. Mean temperatures are similar in all the stations. However, fluctuations are more pronounced in Emin Pasha and Speke gulfs located on the southern shores of the lake. In the middle of the lake which is relatively deeper, there is less variability in temperature (Fig 3).

**Table 2:** Temperature (°C) variations at selected sites in Lake Victoria.

Station	Mean± sd	Maximum	Minimum
Emin Pasha gulf	24.79±1.26	28.18	21.93
Mid lake	25.26±0.63	27.45	23.75
Nyanza Gulf	25.24±0.79	28.33	23.25
Sesse islands	25.53±0.79	28.00	23.41
Speke Gulf	25.16±1.16	28.50	21.89

**Fig 4:**
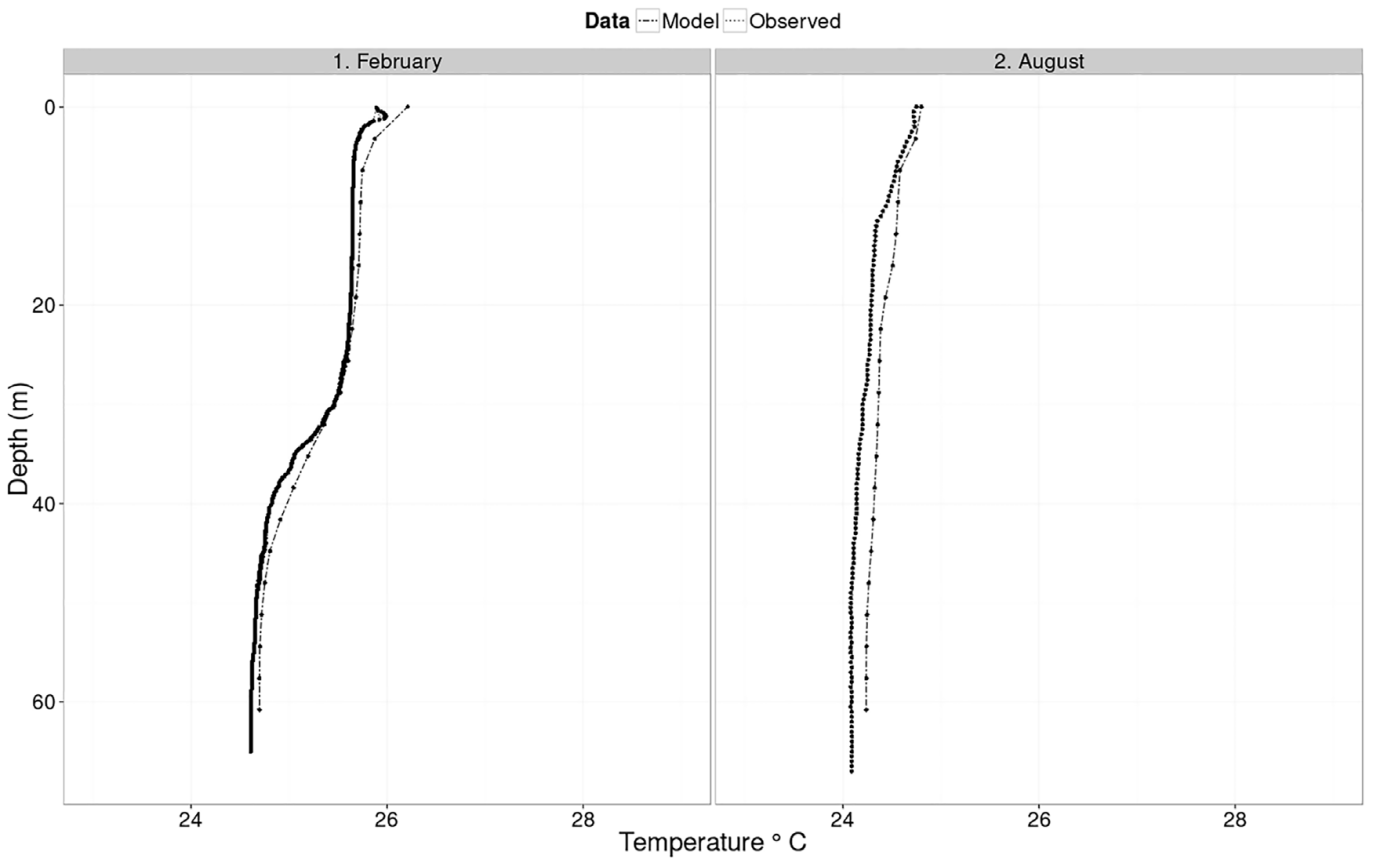
Observed and modeled vertical temperature profiles in Lake Victoria during the months of February and August 2008.

Water temperature exhibits seasonal oscillations over the model domain with periods of clear differences between surface and bottom layers intercepted with spells of near isothermal conditions (Fig 3). Isothermal conditions generally appear to start in either May or June and extend to July or August in different years of the model domain. Surface water temperatures are highest during the months of May and June. Temperatures then decline to an annual low in the months of August and October in the surface and bottom layers respectively. Thereafter the temperature steadily rises before taking a downward turn in March and May for the surface and bottom layers respectively. The two month lag in either warming or cooling of the bottom layers leads to stratification when the two processes are out of sync. Fig 4 shows modeled and observed vertical temperature profiles in the middle of the lake during the months of February (stratified) and August (isothermal) 2008. Differences in RMSE values between modeled and observed temperature profiles are 0.73 and 0.22 in February and August respectively, and generally the modeled temperature closely matches the observed profile.

**Fig 5:**
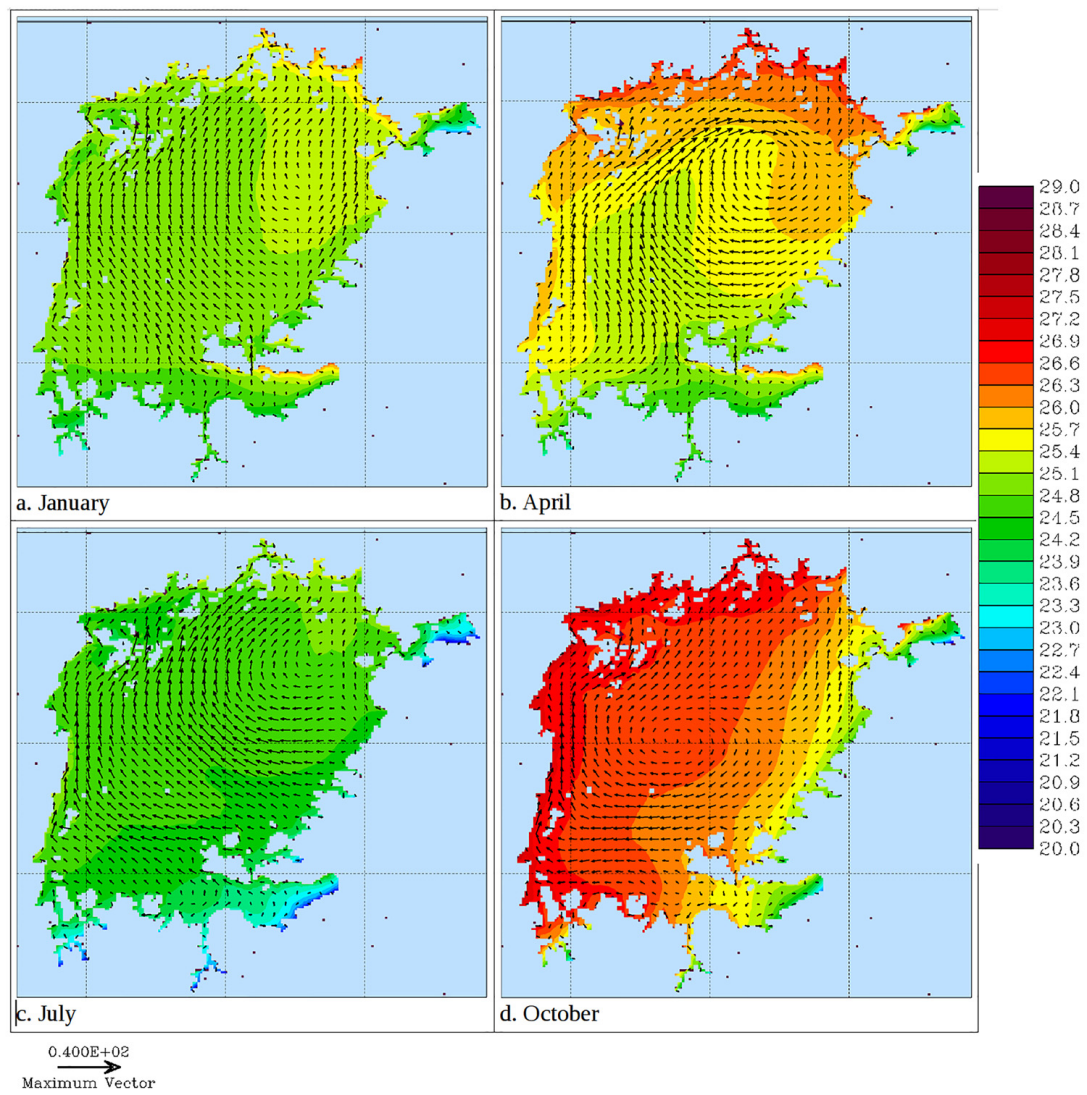
Modeled 20 day averaged near surface (5 m) temperature (shaded) and currents (vectors) in Lake Victoria in different seasons of 2008.

**Fig 6:**
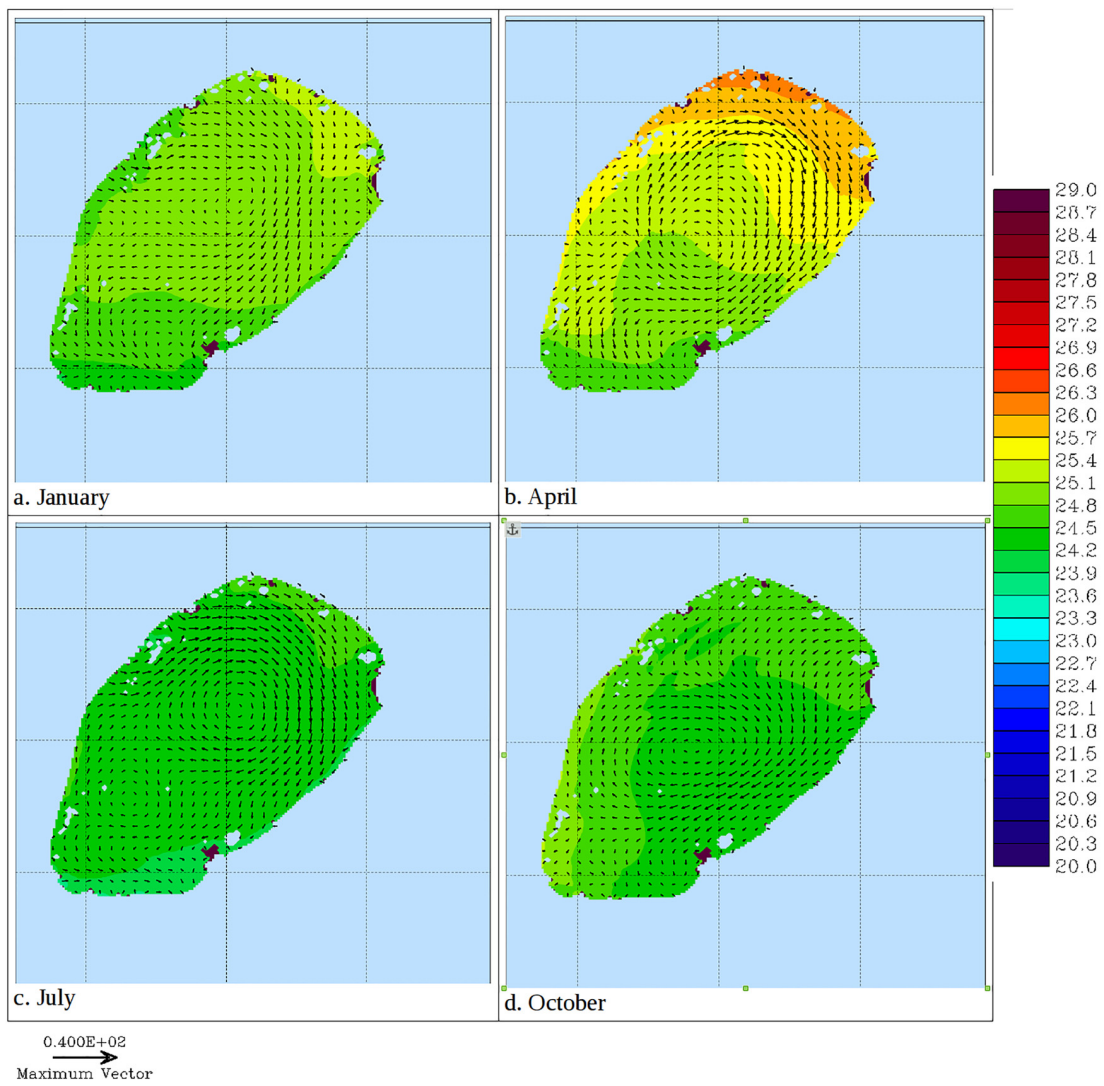
Modeled 20 day averaged temperature (shaded) and currents (vectors) at 35 m in Lake Victoria in different seasons of 2008.

**Fig 7:**
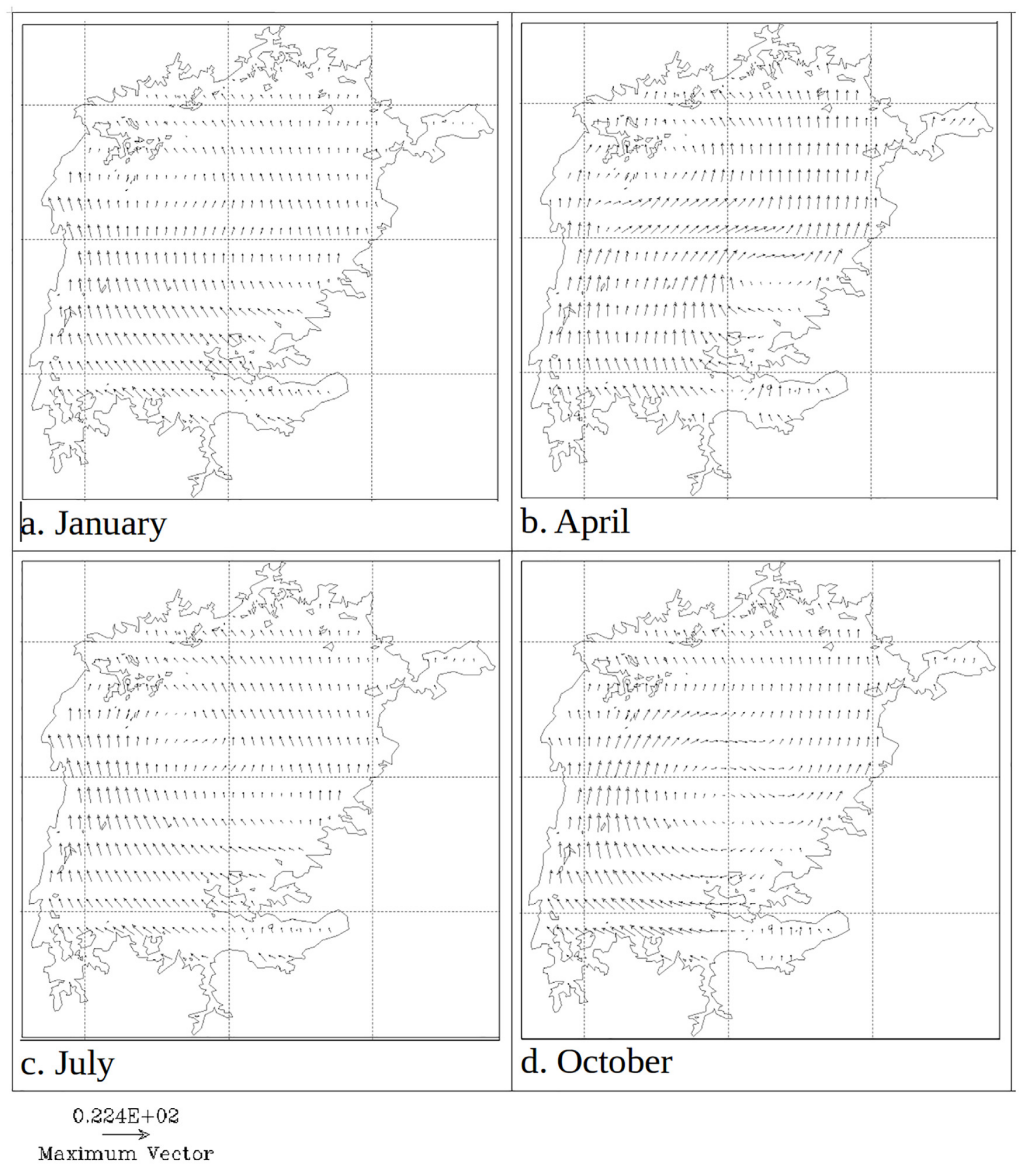
Modeled 20 day averaged wind curl (vectors) in Lake Victoria in different seasons of 2008.

Figs 5–6 show temperature and water circulation patterns (currents) at 0.5 m and 35 m depths respectively in different months of 2008. The modeled circulation at the surface follows the general pattern of the wind curl depicted in Fig 7. In January, 20 day average surface temperatures are generally uniform throughout the lake with the exception of Nyanza Gulf. Surface currents generally flow northwards in most of the open lake (Fig 5a). In April, surface temperatures are higher in the northern near-shore areas relative to the rest of the lake. During this period, surface currents flow in a gyre formation whose center is drawn towards the warmer regions in the north (Fig 5b). Surface temperatures are generally lowest in July and August. This is especially so in the south. As the surface temperature decreases, the gyre circulation disappears and the currents flow northwards (Fig 5c). The temperatures are highest in the month of October, with the western and northern shorelines being relatively warmer than the rest of the lake. A gyre circulation pattern is also evident during this period, and like in April, its center is drawn towards the warmer region (Fig 5d).

**Fig 8:**
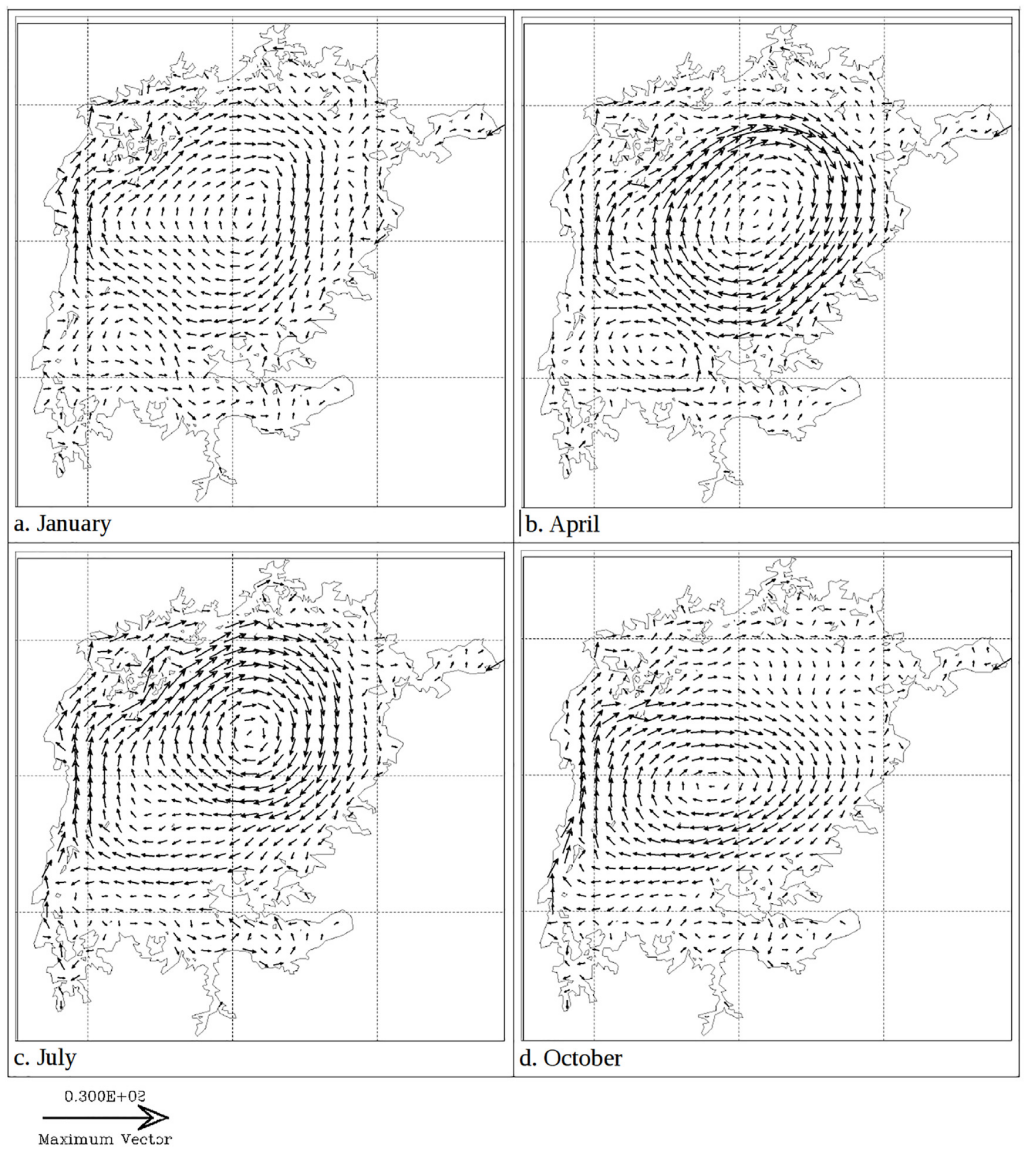
Modeled 20 day verically averaged currents (vectors) in Lake Victoria in different seasons of 2008.

**Fig 9:**
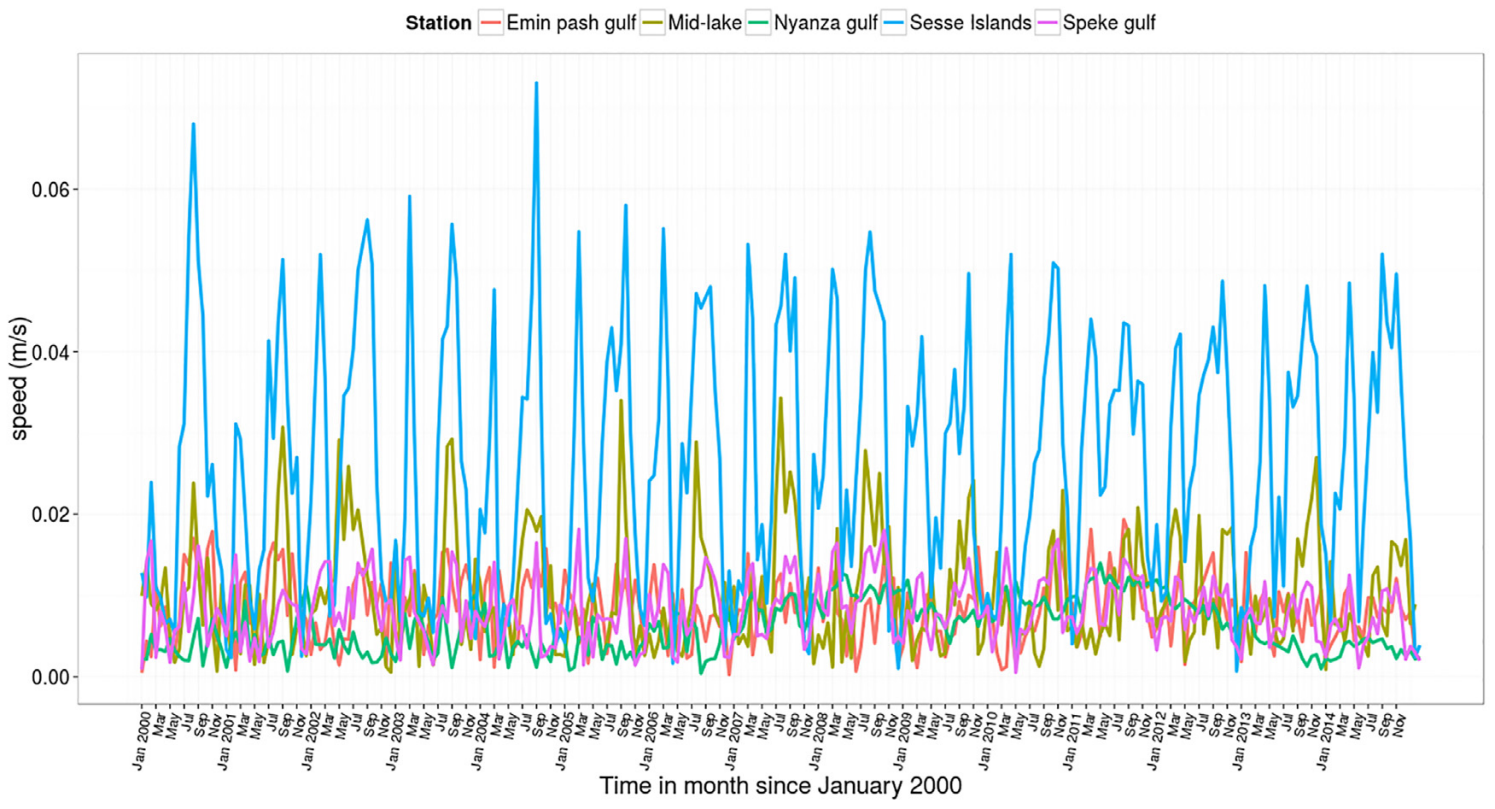
Modeled 20 day currents velocities from the year 2000–2014 at selected sites in Lake Victoria.

At 35 meters depth, the trend is similar to the surface except that the temperatures are a bit lower and they hit their lowest levels two months later than at the surface. Water circulation takes on various patterns at different times of the year (results not shown). Largely it is a one gyre pattern but two or more gyres are manifest at different times of the year. Fig 6c shows two gyre currents at a depth of 35 meters. Gyres are more pronounced in two dimensional (depth averaged) currents (Fig 8). Current velocities are higher in April and July around the gyre formation, especially so in the northern part near Sesse Islands (Fig 9). High current velocities also prevail in the middle of the Lake. However relatively slow flow rates are observed within the three major gulfs of Lake Victoria.

**Fig 10:**
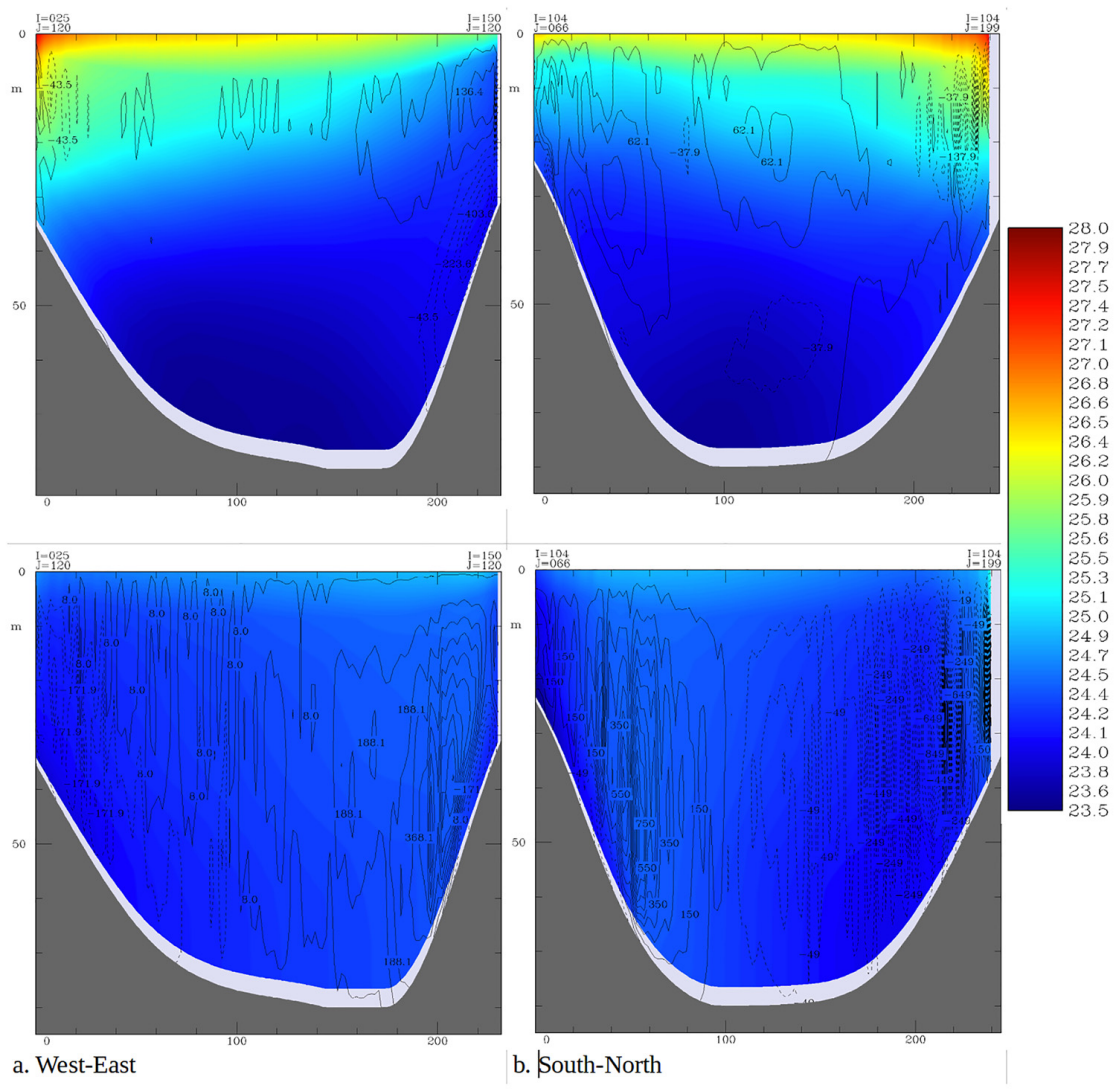
The modeled daily averaged water temperature (shaded, in °C) and vertical velocity (contour, in cm/day and positive upward) in Lake Victoria in stratified (upper row) and isothermal (lower row) conditions in 2008. The west—east section (left) is along 1.0°S while the south—north section (right) is along 33.2°E.

As expected, model results indicate that vertical water velocities are higher during the isothermal period as compared to the stratified period (Fig 10) in both west-east and south-north cross sections. In the stratified period, when vertical velocities are lower, there seems to be minimal exchange of water across the thermocline. On the other hand there is water circulation throughout the water column in the mixed season. During the stratified period there appears to be downwelling at the western and eastern ends of the lake with minimal upwelling above the thermocline at the center of the lake. Generally there is net upward water circulation in the south and some downwelling in the north. During the mixed (isothermal) period, there is upward and downward movement of water in the east and west respectively (Fig 10a). Similarly there is upwelling in the south and downwelling in the north (Fig 10b).

## Discussion

The present study used ROMS to simulate water temperature and circulation patterns in Lake Victoria. Model results are compared to available spatial-temporal temperature data collected during the 2000–2001 and 2005–2014 lake-wide hydro-acoustic surveys. Modeled temperature trends both at the surface and the bottom fit well to observation data. This is especially so for data collected during the 2005–2014 surveys. At the beginning of the simulation (2000–2001), predictions deviate from data points because of the spin-up time the model requires to reach a state of equilibrium under the applied forcing. This is more pronounced in the deeper regions that require a longer spin-up time. Comparison of simulated and observed vertical temperature profiles in the middle of the lake are also presented with RMSE showing minimal inconsistencies between the two profiles. Additional model validation is derived from the general conformity of surface water currents and wind curl fields. Results show that the water column is thermally stratified most of the time, only experiencing complete vertical mixing once a year during the months of June to July. Isothermal conditions last up to August in some years. The seasonal changes in water column temperature profiles coincide with those observed in other studies conducted in Lake Victoria [6, 16–18]. The ability of ROMS to mimic seasonal changes in surface and vertical water temperature profiles confirm the reliability of the simulations. It therefore presents a unique opportunity of exploring other physical processes, mainly water circulation patterns, in Lake Victoria. Water circulation is important in influencing water quality, distribution of nutrients, movement of plankton biota and ultimately influences other ecosystem dynamics [5].

Thermal stratification occurrs when surface waters warm up faster than the underlying layers. This leads to the formation of a thermocline across which there is little exchange of water. Breakdown of the thermocline can be attributed to a number of factors. As the surface waters start to cool down, the lake becomes isothermal and stratification breaks down. Changes in surface temperatures correspond well with fluctuations in solar radiation implying that the latter plays a crucial role in stratification and mixing of the water column of Lake Victoria. The long rains of March through May could also play a role in reducing surface temperature given that precipitation accounts for up to 80 percent of the lake’s water inputs. Currents resulting from increased wind stress accelerate the process of vertical mixing and the eventual breakdown of the thermocline, an observation also made by MacIntyre (2014) [6].

Currents take several forms throughout the year. When water temperatures at the surface are nearly uniform spatially, a general northward flow is sustained in the open lake. This pattern has also been reported in Lake Victoria by MacIntyre (2014) [6]. However, when surface waters in some regions are warmer, gyres prevail. Gyres range from a single one, that engulfs most of the lake and generally flows clockwise at the surface, to two that spin in opposite directions. Sometimes several short-lived small gyres drive water circulation in the lake. Anyah (2009) [4] also reported gyre formation in Lake Victoria. Deeper water masses exhibit a counter flow to surface water layers. Counter currents occur at the fringes of gyres. A consequence of this, is the formation of upwelling and downwelling regions that shift spatially due to the changing of circulation patterns. Upwelling causes a motion of dense, cooler, nutrient-rich water towards the surface, replacing the warmer, nutrient-depleted surface water. The nutrient-rich upwelled water stimulates the growth and reproduction of primary producers (phytoplankton). Knowledge of the location and timing of upwelling obtained from this study will help predict presence of high levels of primary productivity and thus fishery production in Lake Victoria. MacIntyre (2014) [6] observed that upwelling occurs on the basin scale in response to increased southerly winds over Lake Victoria. Vertical velocities in both the west-east and south-north cross-sections depict the influence of stratification on water circulation. The velocities are minimal during the stratified period and water movements are limited to either side of the thermocline. Lack of exchange of water between surface and bottom layers explain the development of anoxic conditions in the latter that are prevalent during the stratified period [6, 10].

Water circulation patterns in the three gulfs (Nyanza, Speke, and Emin Pasha) are localized. They are characterized by relatively slow water current velocities, probably because they are sheltered and shallow (especially Nyanza which is connected to the main lake via a narrow channel). This could inhibit water exchange between the gulfs and the open lake. The gulfs coincidentally host major river mouths and large urban settlements through which nutrients, sediments and pollutants enter the lake. Due to the little flushing with water from the lake, these regions are limnologically different from the open lake and are particularly more turbid and eutrophic.

Circulation in Lake Victoria is mainly driven by differential heat fluxes, wind stress and bottom terrain. Spatial variability in latent heat fluxes creates conditions conducive for horizontal convective circulation [10, 19]. The difference in water density as a result of temperature variations causes gravity currents at the surface [20].

## Conclusion

Information on water temperature and circulation patterns in Lake Victoria has been limited prior to this study. Reliability of the generated model is reinforced by its ability to fit to observational spatial-temporal temperatures trends as well as replicating vertical temperature profiles in the lake during both mixed and stratified periods. Simulated data show that the water column exhibits annual cycles of thermal stratification (September–May) and mixing (June–August). Vertical velocities are low during the stratified period and are localized to either side of the thermocline. This explains the low oxygen levels that occur in deep waters during the stratified period.

Surface water currents in Lake Victoria take on different patterns ranging from a lake-wide northward flow to gyres that vary in size and number. An underflow is a constituent of the circulation leading to the formation of upwelling regions that shift in time and space. Current velocities are highest at the center of the lake and also in the western inshore waters indicating enhanced water circulation in those areas. On the contrary, there seems to be little exchange of water between the major gulfs (especially Nyanza) and the open lake, a factor that could be responsible for the different water quality reported in those regions [6]. Information on the location and timing of upwelling and vertical mixing obtained from this study will help predict presence and location of high levels of primary productivity and thus fishery production in Lake Victoria.

Temporal and spatial differences in both water temperature and circulation (currents), are attributable to a range of atmospheric forcing effects and lake terrain. Findings of the present study enhance our understanding of the physical processes of the Lake Victoria and consequently form a basis for future comprehensive ecosystem studies that quite often require physical hydrodynamic components. The developed model also forms a basis for which future observations could be made to refute or corroborate findings of this study.

## Supporting Information

S1 Files Shape files for a map of Lake Victoria in East Africa, its major rivers and bathymetry. (ZIP)
